# Studying the Association between Antibiotic Resistance Genes and Insertion Sequences in Metagenomes: Challenges and Pitfalls

**DOI:** 10.3390/antibiotics12010175

**Published:** 2023-01-14

**Authors:** Lucie Galiot, Xavier C. Monger, Antony T. Vincent

**Affiliations:** 1 Département Des Sciences Animales, Faculté Des Sciences de L’agriculture et de L’alimentation, Université Laval, Quebec City, QC G1V 0A6, Canada; 2 Institut de Biologie Intégrative et Des Systèmes, Université Laval, Quebec City, QC G1V 0A6, Canada

**Keywords:** microbiome, bacteria, antibiotic resistance genes, insertion sequences, bioinformatics

## Abstract

Antibiotic resistance is an issue in many areas of human activity. The mobilization of antibiotic resistance genes within the bacterial community makes it difficult to study and control the phenomenon. It is known that certain insertion sequences, which are mobile genetic elements, can participate in the mobilization of antibiotic resistance genes and in the expression of these genes. However, the magnitude of the contribution of insertion sequences to the mobility of antibiotic resistance genes remains understudied. In this study, the relationships between insertion sequences and antibiotic resistance genes present in the microbiome were investigated using two public datasets. The first made it possible to analyze the effects of different antibiotics in a controlled mouse model. The second dataset came from a study of the differences between conventional and organic-raised cattle. Although it was possible to find statistically significant correlations between the insertion sequences and antibiotic resistance genes in both datasets, several challenges remain to better understand the contribution of insertion sequences to the motility of antibiotic resistance genes. Obtaining more complete and less fragmented metagenomes with long-read sequencing technologies could make it possible to understand the mechanisms favoring horizontal transfers within the microbiome with greater precision.

## 1. Introduction

Antibiotics are used to control bacterial diseases in humans and animals. The overuse of antibiotics in various spheres of human activity has led to evolutionary selection and an increase in bacterial strains resistant to antibiotics. This has made it more difficult to treat bacterial infections [1]. Animal-based food production consumes over 75% of the total antibiotics consumed in Canada [1]. In recent decades, antibiotic-resistant bacteria have been on the rise, causing major food safety issues, particularly when these bacteria are pathogenic [2]; humans can become infected either through direct contact or consumption of contaminated food [1,2,3]. A report from Murray (2022) [4] stated that in 2019, 4.95 million deaths worldwide were associated with bacterial resistance; 1.27 million of those were directly due to antibiotic-resistant bacteria. In Canada, in 2019, over 25% of bacteria that caused human infection were resistant to at least one antibiotic [1].

Although bacteria can develop resistance to antibiotics when mutations occur in the parts that are targets of antimicrobial compounds, they can also have antibiotic-resistance genes (ARGs) that encode proteins dedicated to resistance. Typically, these proteins interact with antibiotics to modify them, to make them ineffective or to alter the inflow and outflow of antibiotics from bacterial cells [5]. The gut microbiome is an important reservoir of ARGs, with a known transfer of ARGs from gut microbes to pathogens [6]. The majority of ARG transmission occurs via mobile genetic elements that can be exchanged between bacterial species [7]. There are two types of mobile genetic elements: intercellular elements are transmissible from cell to cell, as with plasmids and bacteriophages; while intracellular elements are transposable elements that cannot be transferred by themselves, and include transposons, insertion sequences (ISs), integrons, and introns [8,9].

ISs are short elements typically composed of only one or two open reading frames surrounded by terminal motifs [10]. The open reading frames encode transposase, which catalyzes DNA excision/insertion of the IS and regulatory proteins [10]. Insertion sequences could also form composite transposons that involve two flanking ISs, responsible for the cleavage and insertion of larger regions of DNA, which may carry ARGs and other genes [11]. During an exposure to antibiotics, the relationship between ISs and ARGs was demonstrated by their increased association on bacterial genomes [11]. In addition, identification of highly variable composite transposons has shown promise for determining potential novel resistance [12]. However, it does not seem that all ISs have a significant association with ARGs, and the underlying mechanisms are not well understood.

To study relationships within the microbiome between ARGs and ISs, two public datasets were used. The first made it possible to analyze the effects of different antibiotics (amoxicillin, doxycycline, and ciprofloxacin) in a controlled mouse model [13]. The second dataset came from a study of the differences between conventional and organic/natural farms in cattle [14] and therefore made it possible to verify the hypothesis in a model representative of the field. While several associations could be made between ARGs and ISs, certain technical challenges to be addressed, and pitfalls to avoid, in the interpretation of the results remain.

## 2. Results

### 2.1. Associations between ARGs and ISs in a Mouse Model

**Figure 1:**
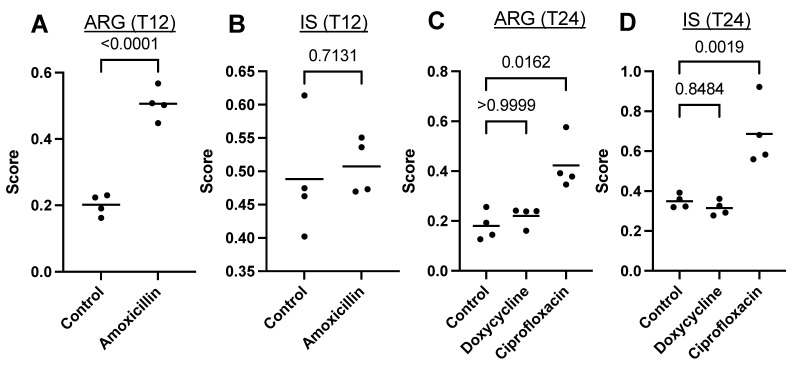
Quantification of ARGs (**A**,**C**) and ISs (**B**,**D**) for different antibiotic treatments. T12 and T24 indicate that the treatments were either administered for 12 h or 24 h. *p* values are shown for all comparisons versus controls.

The hypothesis that there is a relationship between antibiotic resistance genes and insertion sequences was first verified using a public dataset that allowed us to study the effects of three antibiotics on the gut microbiome of mice: amoxicillin (administered for 12 h), ciprofloxacin, and doxycycline (administered for 24 h) [13]. First, ARGs and ISs were quantified using sequencing reads using the bioinformatics tool MetaProtMiner (Figure 1). There was a significant increase in ARGs for two of the three treatments (amoxicillin and ciprofloxacin), as expected. The doxycycline treatment caused no significant increase in the number of reads mapped to ARGs. For ISs, only the ciprofloxacin treatment caused a significant increase.

**Figure 2:**
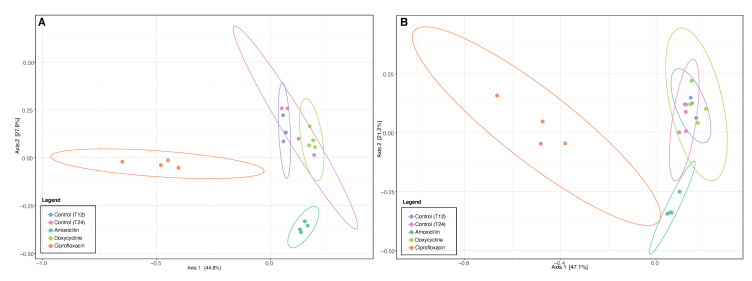
PCoAs showing the distribution of samples according on the presence and amount of ARGs (**A**) and ISs (**B**) in the samples. Samples are colored according to the antibiotics used (control, amoxicillin, ciprofloxacin, and doxycycline). The ellipses represent a 95% confidence interval.

Analyzing the distribution of the samples in greater detail, with PCoAs based on ARGs and the ISs (Figure 2), we observed that the samples from mice treated with amoxicillin had a different distribution from the control samples, similarly to ciprofloxacin. Since no difference was observed with the global quantification of samples from mice treated with amoxicillin for ISs (Figure 1), this means that some ISs increased, while others decreased, in a similar ratio. Therefore, at this stage, amoxicillin and ciprofloxacin seemed to alter the populations of ARGs and ISs, while doxycycline had no significant effect. There would therefore be an antibiotic-dependent effect.

**Figure 3:**
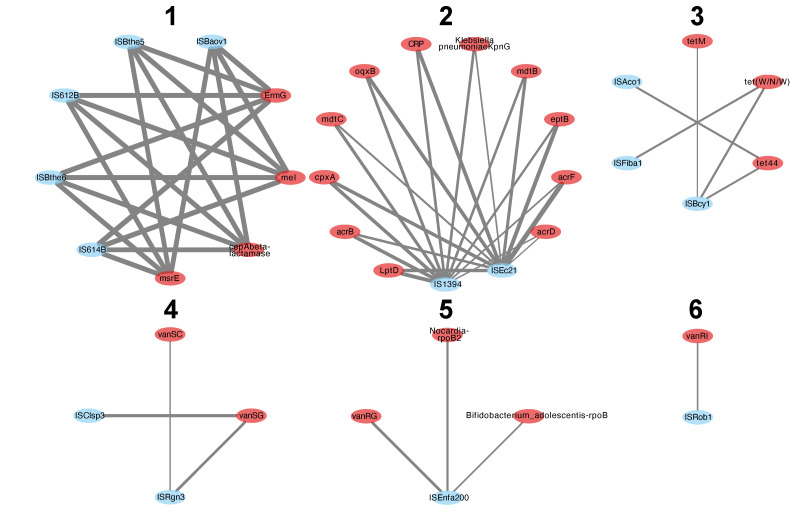
Correlations between ARGs (red) and ISs (blue). The six clusters are numbered from 1 to 6. The thickness of the lines reflects the strength of the correlations between the elements.

A correlation analysis between ISs and ARGs that included all the samples showed that there were significant correlations (*p* < 0.01) distributed in six groups (Figure 3). Among the six groups of correlations between IS and ARGs, two stood out for their high density. Group 1 consisted exclusively of ISs found in bacteria of the genus *Bacteroides*, according to the ISfinder database [15]. For Group 2, IS*1394* is found in *Pseudomonas alcaligenes*, while IS*Ec21* is in *Escherichia coli*. Although it was tempting to conclude that antibiotic treatments increased the correlations between ISs and ARGs in these bacteria, it was important to verify whether these correlations could have been caused by a large fluctuation in the population of these bacteria.

**Figure 4:**
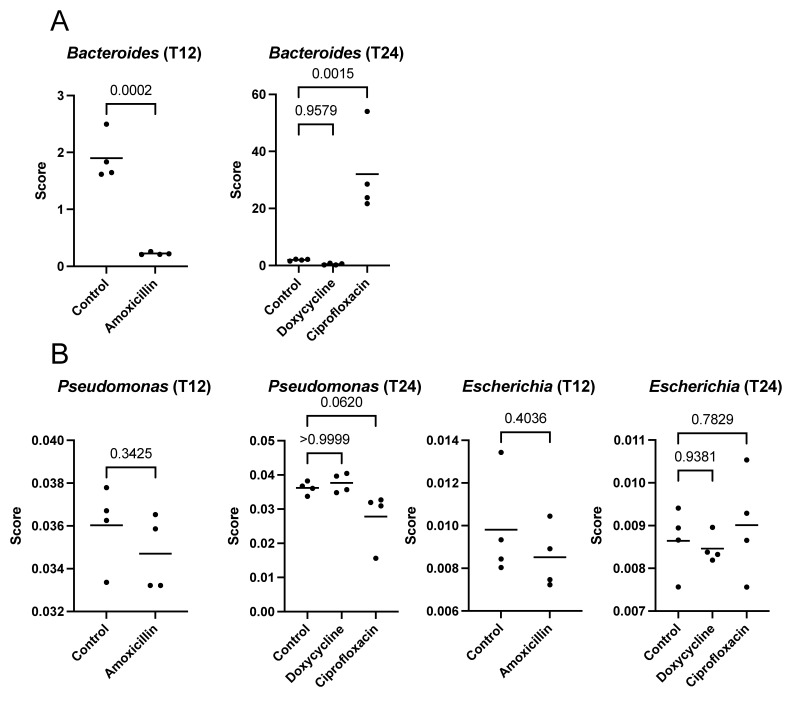
Fluctuation of populations of *Bacteroides* (**A**), and *Pseudomonas* and *Escherichia* (**B**) for T12 and T24. *p* values are shown for all comparisons versus controls.

There was indeed a strong fluctuation in the population of *Bacteroides* for samples from mice treated with amoxicillin and ciprofloxacin (Figure 4A). In fact, the fluctuation was so strong that it was impossible to determine whether there was a real and direct correlation between the ARGs and the ISs for these samples. However, no significant fluctuation was found for *Pseudomonas* and *Escherichia* (Figure 4B). This suggests that the correlations between ARGs and ISs could not be fully explained by a modulation of the population of these two bacteria. In fact, the results suggest that subpopulations of these bacteria containing specific combinations of ISs and ARGs may have replaced subpopulations without these combinations.

### 2.2. Associations between ARGs and ISs in a Real Agricultural Context

To better understand the impact of agricultural practices on the spread of antimicrobial resistance, a public dataset was used to facilitate the study of the differences between conventional and organic/natural farms in dairy and feedlot cattle, and to investigate the relationships between ARGs and ISs [14]. This dataset made it possible to study a ruminant model in addition to mice, which are monogastric and therefore have very different microbiomes.

**Figure 5:**
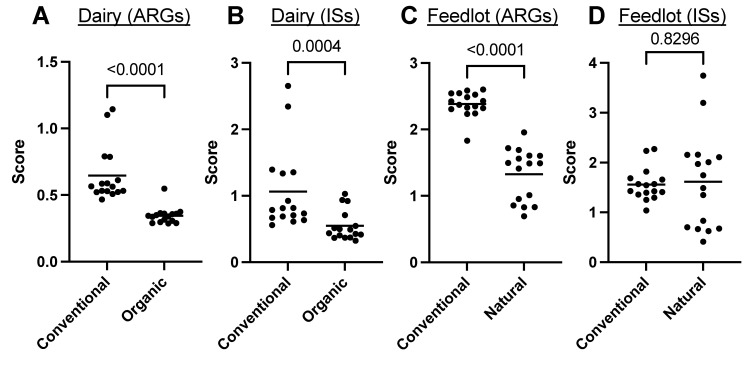
Quantification of ARGs (**A**,**C**) and ISs (**B**,**D**) for dairy cows (**A**,**B**) and animals reared in a feedlot (**C**,**D**). *p* values are shown for each of the comparisons.

By determining the overall composition of ARGs using the MetaProtMiner scores, it was possible to see that the organic/natural practices significantly decreased the presence of ARGs (Figure 5A,C). ISs were significantly lower in the organically raised dairy cows than in conventionally raised dairy cows (Figure 5B). However, no significant difference was observed for natural vs. conventional feedlot cattle (Figure 5D).

**Figure 6:**
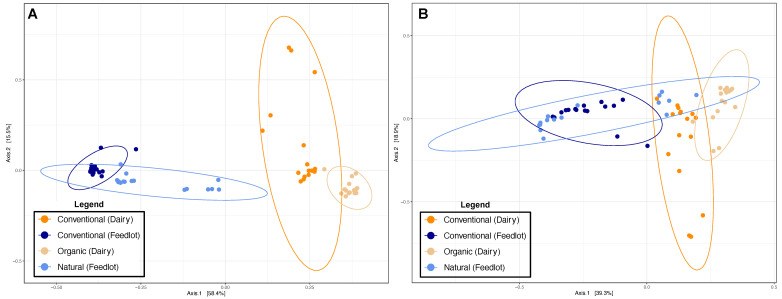
PCoAs showing the distribution of samples according to ARGs (**A**) and ISs (**B**). Samples are colored according to the production type and practice (dairy, feedlot, conventional, and organic/natural). The ellipses represent a 95% confidence interval.

The distribution of samples according to ARGs and ISs was verified using PCoAs (Figure 6). For both ARGs (Figure 6A) and ISs (Figure 6B), the samples tended to group together depending on whether the animals came from a dairy or a production feedlot. For each of the practices (conventional or organic/natural), there was also a clear separation of the samples according to the ARGs (Figure 6A). For the ISs (Figure 6B), a separation of the samples from dairy cows was visible, while samples from animals in the feedlot did not seem to be clearly grouped according to the different practices (conventional or natural). These results are similar and consistent with the overall scores of the samples found by MetaProtMiner (Figure 5).

**Figure 7:**
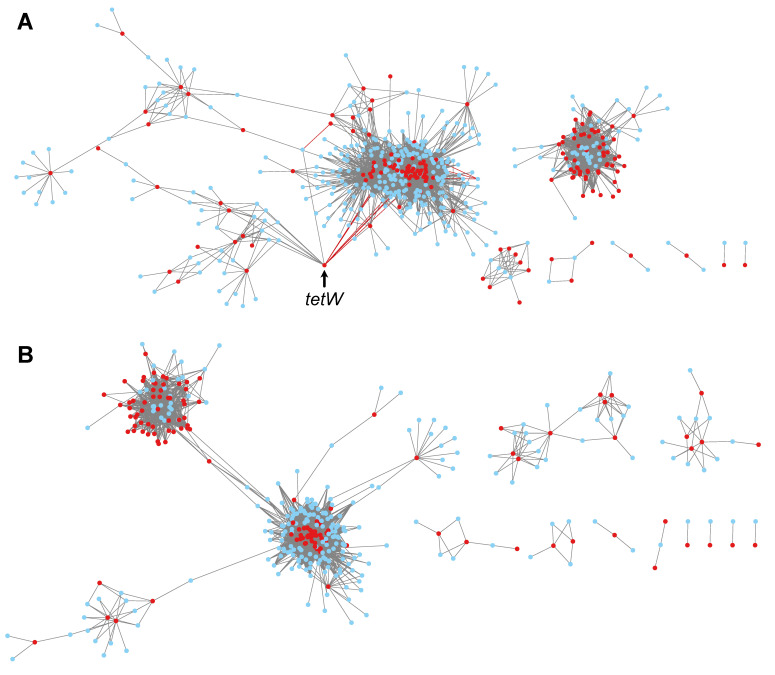
Correlations between ARGs (red circles) and ISs (blue circles) for data from dairy cows (**A**) and feedlot cattle (**B**). For dairy cows, the red links indicate the negative correlations with the *tetW* gene (shown by the arrow).

An analysis of the correlations between ARGs and ISs showed that ARGs and ISs correlated in a complex network, both for dairy cows (Figure 7A) and for feedlot cattle (Figure 7B). While the correlation network produced with data from mice contained relatively few nodes and had a high density of these, the networks with cattle, on the contrary, had many nodes and were less dense. For example, the normalized network density value, which represents the propensity of nodes in a network to be strongly or weakly connected, was 0.282 for the network with data from mice, 0.059 for dairy cattle, and 0.080 for feedlot cattle.

All of the correlations between ARGs and ISs with data from mice were positive; most correlations found with cattle data were also positive. Only 11 correlations were negative in dairy cows. Of these 11 negative correlations, seven (~64%) involved the *tetW* gene providing resistance to tetracycline in several anaerobic intestinal and ruminal bacteria [16]. Interestingly, the *tetW* gene was also implicated in several positive correlations with other ISs from various bacteria (Table 1). Among the seven negative correlations involving *tetW*, five were with ISs found in species of the genus *Paracoccus*, a bacterium known to be present in cow’s milk [17].

## 3. Discussion

The rise of antibiotic resistance is a worrying phenomenon in human and veterinary medicine [4]. One of the challenges is that ARGs can typically spread from one bacterial species to another within a microbial population using mobile genetic elements [8]. It is consequently difficult, if not impossible, to contain antibiotic resistance. Therefore, it is crucial to have an integrated approach, of the One Health type, and to better understand the mechanisms that facilitate the mobilization of ARGs.

It is known that certain ISs can facilitate the mobilization of ARGs by forming composite transposons. Moreover, as reviewed elsewhere, ISs can participate in the adaptation of ARGs to new genomic environments by providing promoter regions that allow gene expression [18]. The decrease in costs related to sequencing and the improvement of technologies have made metagenomic studies increasingly accessible, and therefore it is easier to explore microbial communities with great precision. Consequently, it is attractive to use these data to better study the role of ISs in the mobilization of ARGs within a bacterial population. As an example, a recent study identified an ARG transfer network using publicly available metagenomic data [11,19].

In this study, two objectives were pursued. First, to continue to discover associations between ARGs and ISs. Second, to identify challenges in understanding the relationships between ARGs and ISs. To do this, we explored two publicly available metagenomic datasets, one allowing us to investigate the effects of different antibiotics on the mouse microbiome [13] and the other to study the modulation of the microbiome according to different breeding practices in cattle [14].

For samples from mice, it was interesting to note that the compositions of ARGs and ISs differed according to the antibiotic used (Figure 1 and Figure 2). Ciprofloxacin caused the greatest change in both ARGs and ISs, followed by amoxicillin. The structures in ARGs and ISs therefore seemed to be dependent on the antibiotic used.

However, although ARGs and ISs fluctuated depending on the antibiotic, the crucial point is whether there were correlations between these two entities, suggesting that one could favor the other.

It was possible to identify significant correlations between ARGs and ISs for data from mice (Figure 3). These correlations, visualized in the form of a network, were distributed in several clusters, including two main ones grouping together most of the correlations. It was tempting to see in the correlations a co-selection resulting from a molecular mechanism favoring the ARGs (such as the formation of composite transposons, or the overexpression of the ARGs). However, it was crucial to verify whether the correlations between ARGs and ISs could be explained by bacterial fluctuations.

One of the two main clusters was composed of ISs found in *Bacteroides*, while the other was composed of IS from *E. coli* and *Pseudomonas* sp. A taxonomic analysis of the samples made it possible to demonstrate that there was, indeed, a very strong fluctuation of *Bacteroides* caused by the antibiotics (Figure 4), which had already been demonstrated by the study which produced the data [13]. However, no significant difference was found for *Escherichia* and *Pseudomonas*.

It is difficult to estimate the true value of the correlations between ARGs and ISs from *Bacteroides*, since the ISs may have been indirectly selected, as they were in the same cells as the ARGs, which could have allowed adaptation to these bacteria. However, for *E. coli* and *Pseudomonas*, since the correlations between the ARGs and the ISs were significant, but not the fluctuation of the population, it is possible to suggest that some of these correlations may have been the origin of a molecular mechanism allowing certain cells of these bacteria to replace those which did not have the conditions to survive.

A second model with dairy and feedlot cattle, according to different real farming practices (conventional or organic/natural), was used to study the relationships between ARGs and ISs. As expected, a higher number of ARGs were detected in samples from conventionally reared animals than those from organically/naturally reared animals (Figure 5). Similarly, more ISs were found in dairy cows reared on conventional farms. Interestingly, no significant difference was found for ISs between different practices for feedlot cattle. The PCoAs representing the distribution of the samples according to the populations of ARGs and ISs showed the same phenomenon (Figure 6), i.e., a difference of ARGs and ISs for dairy cows according to agricultural practice, whereas for animals in a feedlot, a separation of the samples was only visible for the ARGs. It is also interesting to note that the PCoAs showed that dairy cows and feedlot cattle had intrinsically different compositions of ARGs and ISs. This, therefore, demonstrated that it would be difficult to extrapolate conclusions found for one of the groups to the other.

An analysis of the correlations between ARGs and ISs revealed a much more complex network than in the mouse model, both for dairy cows (Figure 7A) and for feedlot cattle (Figure 7B). Several factors may possibly explain this complexity. Ruminants have a markedly different microbiome, with a higher richness than monogastrics such as mice [20]. Compared to the mice, which were part of a controlled experiment [13], the cattle came from farms. The animals were therefore subject to greater variations in infections, the drugs used, and environmental fluctuations. Finally, the number of animals was higher in the experiment in cattle than that with mice. It is therefore possible that certain correlations became statistically stronger and therefore significant.

Although it was impossible to analyze the networks as finely as for the experiment with the mice, it was possible to observe different groups of ARGs/ISs interactions, which suggests that there were indeed relations between these two entities. It is also interesting to note that negative correlations were detected in the dairy cow samples (Table 1). Several of the negative correlations involved the *tetW* gene, providing resistance to tetracycline, an antibiotic widely used in agriculture [21]. Almost all negative correlations with *tetW* were with ISs found in the bacterial genus *Paracoccus*. Two hypotheses can be proposed to explain these negative correlations. First, it is possible that *Paracoccus* is refractory to acquiring *tetW* or the vectors where *tetW* is located. Second, the *tetW* gene may favor certain bacteria that compete with *Paracoccus*.

**Figure 8:**
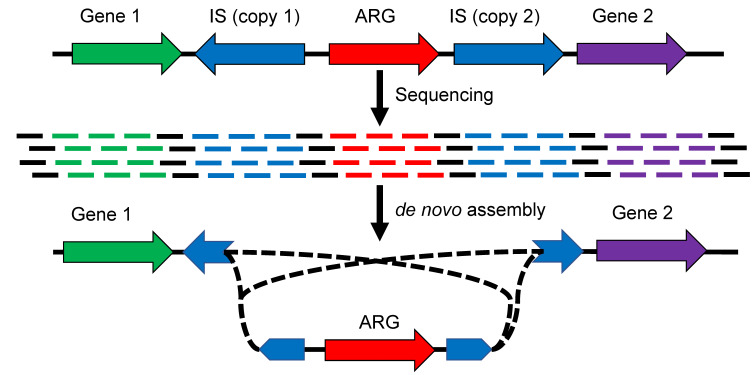
Schematization of the challenge of studying the relationships between an ARG and two ISs following de novo assembly.

Ultimately, the study of ARG and IS relationships should be performed by validating the proximity of the two entities on the different assembled sequences. However, most shotgun metagenomics data are generated with very high-throughput but short-read (~150 bp) sequencers. It is known that ISs, as any repeated genomic elements, can induce breaks in the assembly if they are longer than the reads [22], which is generally the case (Figure 8). Due to the complexity and diversity of metagenomes, ARGs can also be duplicated and cause breakage in the assembly [23]. Therefore, investigating the proximities between ISs and ARGs in this type of data can induce large biases and lead to erroneous conclusions.

Long-read sequencing technologies, such as those from PacBio and Oxford Nanopore, are becoming more efficient and affordable. Two tools, mbin [24] and nanodisco [25], even make it possible to use the epigenetic data produced natively by these technologies to group together mobile DNA elements, such as plasmids, with bacterial chromosomes. The use of long-read technologies could overcome the limitations of short-read technologies and allow researchers to precisely study the relationships between ARGs and ISs in their genomic context, as well as to better understand the role of the microbiome in the mobility of ARGs in general.

## 4. Conclusions

The detection of the molecular determinants that are responsible for resistance to antibiotics is essential to establish effective monitoring protocols and to combat the increase in resistance to antibiotics in the long term. The present study investigated the relationships between ISs and ARGs at the microbiome level of animals under different conditions (antibiotic treatments or production systems). Although we found correlations between certain ISs and ARGs, helping us to better understand the mechanisms favoring gene exchanges in a bacterial population, several challenges remain. One of these is that the inherent complexity of metagenomes makes it difficult to obtain high quality complete sequences. The accessibility and improvement of long-read sequencing technologies (PacBio or Oxford Nanopore) could partly overcome this problem and considerably increase the precision of analyses. The portability of certain systems, such as Oxford Nanopore’s MinION, could even make possible rapid detection in the field for real-time diagnosis of the ability of antibiotic resistance genes to spread, thus allowing increased surveillance of risky environments.

## 5. Materials and Methods

The two datasets were downloaded from the NCBI SRA database. One recorded the effect of different antibiotics on the mouse microbiome (SRA: PRJNA504846). The other documented the impact of breeding practices on the microbiome of cattle (SRA: PRJNA379303). Sequencing reads were first filtered with the OpenGene tool fastp version 0.23.1 [26]. Subsequently, the corresponding host reads were removed by mapping all reads against the genome sequences of *Mus musculus* (GenBank: GCF_000001635.27) or *Bos taurus* (GenBank: GCF_002263795.1), using a combination of Bowtie 2.4.4 [27] and SAMtools 1.13 [28].

The cleaned reads were used to analyze the presence of ARGs and ISs in the datasets using MetaProtMiner, a tool specially designed for the present study (https://github.com/ATVincent/MetaProtMiner), accessed on 28 December 2022. First, reads were mapped to a specialized protein database with Diamond version 2.0.13’s blastx function [29]. The CARD [30] and ISfinder [15] databases (as distributed by Prokka [31]) were used for ARGs and ISs, respectively. Subsequently, for each element (ARG or IS), the number of reads that mapped to this element were divided by the length of the element and by the total number of reads in the dataset. This reduces the bias, since a larger element will necessarily have more mapped reads than a smaller element, even with an equal quantity in the dataset, and this makes it possible to compare elements between datasets with different sequencing depths. Subsequently, the standardized value of each of the elements was multiplied by 1,000,000, to avoid having values that were difficult to work with due to being too small. Finally, MetaProtMiner provided a table with the different standardized elements and an overall score (the sum of the different elements), to give an idea of the global quantity of elements in the analyzed dataset. For the present study, only alignments that presented an identity percentage of 90% and a coverage of 75% were considered.

Principal coordinates analysis (PCoA) and graphical outputs were generated via R software version 4.1.1 with package phyloseq v.1.36 [32]. Comparisons between global scores were made in PRISM 9 using an unpaired *t*-test, Mann–Whitney test, Kruskal–Wallis test, or one-way ANOVA, depending on the number of samples and whether the data were normally distributed or not (d’Agostino–Pearson test or Shapiro–Wilk test). Correlations were analyzed using the ccrepe package version 1.7.0 [33]. Visualizations of correlations were made using Cytoscape version 3.9.1 [34] with a *p*-value threshold of 0.01 and a score of 0.75. Taxonomic analyzes were performed with Kaiju version 1.8.2 [35] and the database nr.

## Figures and Tables

**Table 1:** Correlations involving the *tetW* gene in the dairy cow dataset.

IS	Family	Score	*p*-Value	Bacteria
IS*Cce2*	IS*256*	0.856	3.54 × 10^−11^	*Clostridium cellulolyticum*
IS*Rgn3*	IS*1380*	0.845	6.97 × 10^−10^	*Ruminococcus gnavus*
IS*Stin10*	IS*200*/IS*605*	0.828	4.52 × 10^−10^	*Streptococcus iniae*
IS*Dha13*	IS*200*/IS*605*	0.797	7.59 × 10^−8^	*Desulfitobacterium hafniense*
IS*Bf7*	IS*1380*	0.791	6.64 × 10^−9^	*Bacteroides fragilis*
IS*Unb4*	IS*1595*	0.777	5.87 × 10^−9^	*unclassified Bacteria*
IS*Bian1*	IS*5*	0.770	4.09 × 10^−9^	*Bifidobacterium animalis*
IS*Bvu1*	IS*1380*	0.764	2.31 × 10^−9^	*Bacteroides vulgatus*
IS*Cth10*	IS*200*/IS*605*	0.756	1.27 × 10^−7^	*Clostridium thermocellum*
IS*Paes3*	IS*256*	−0.753	9.52 × 10^−8^	*Paracoccus aestuarii*
IS*Pye36*	IS*3*	−0.757	4.01 × 10^−7^	*Paracoccus yeei*
IS*Pye14*	IS*66*	−0.757	1.5 × 10^−7^	*Paracoccus yeei*
IS*Pkr1*	IS*21*	−0.759	7.3 × 10^−8^	*Paracoccus koreensis*
IS*Rsp12*	Tn*3*	−0.765	1.66 × 10^−7^	*Rhizhobium sp.*
IS*1396*	IS*L3*	−0.774	2.71 × 10^−8^	*Serratia marcescens*
IS*Pbe1*	IS*3*	−0.802	1.01 × 10^−8^	*Paracoccus bengalensis*

## Data Availability

The datasets used in this study are available in the SRA database (Mice: PRJNA504846; Cattle: PRJNA379303). The tool MetaProtMiner is available on GitHub (https://github.com/ATVincent/MetaProtMiner (accessed on 28 December 2022)).
